# Different Types of Cell Death Induced by Enterotoxins

**DOI:** 10.3390/toxins2082158

**Published:** 2010-08-11

**Authors:** Chiou-Feng Lin, Chia-Ling Chen, Wei-Ching Huang, Yi-Lin Cheng, Chia-Yuan Hsieh, Chi-Yun Wang, Ming-Yuan Hong

**Affiliations:** 1 Institute of Clinical Medicine, College of Medicine, National Cheng Kung University, Tainan 701, Taiwan; Email: zarbi@mail2000.com.tw (W.-C.H.); sillylilly0518@yahoo.com.tw (Y.-L.C.); dyoushus@yahoo.com.tw (C.-Y.H.); chixuan1225@hotmail.com (C.-Y.W.); myuan@mail2000.com.tw (M.-Y.H.); 2 Institute of Basic Medical Sciences, College of Medicine, National Cheng Kung University, Tainan 701, Taiwan; 3 Department of Microbiology and Immunology, College of Medicine, National Cheng Kung University, Tainan 701, Taiwan; Email: clchen2001.tw@yahoo.com.tw (C.-L.C.); 4 Department of Medical Laboratory Science and Biotechnology, College of Medicine, National Cheng Kung University, Tainan 701, Taiwan; 5 Department of Emergency, National Cheng Kung University Hospital, Tainan 701, Taiwan

**Keywords:** apoptosis, necrosis, enterotoxin, exotoxin, pore-forming toxin, staphylococcal enterotoxin B, staphylococcal alpha-toxin, Panton-Valentine leukocidin, alpha-hemolysin, Shiga toxin, cytotoxic necrotizing factor 1, heat-labile enterotoxin, cholera toxin

## Abstract

The infection of bacterial organisms generally causes cell death to facilitate microbial invasion and immune escape, both of which are involved in the pathogenesis of infectious diseases. In addition to the intercellular infectious processes, pathogen-produced/secreted enterotoxins (mostly exotoxins) are the major weapons that kill host cells and cause diseases by inducing different types of cell death, particularly apoptosis and necrosis. Blocking these enterotoxins with synthetic drugs and vaccines is important for treating patients with infectious diseases. Studies of enterotoxin-induced apoptotic and necrotic mechanisms have helped us to create efficient strategies to use against these well-characterized cytopathic toxins. In this article, we review the induction of the different types of cell death from various bacterial enterotoxins, such as staphylococcal enterotoxin B, staphylococcal alpha-toxin, Panton-Valentine leukocidin, alpha-hemolysin of *Escherichia coli*, Shiga toxins, cytotoxic necrotizing factor 1, heat-labile enterotoxins, and the cholera toxin, *Vibrio cholerae*. In addition, necrosis caused by pore-forming toxins, apoptotic signaling through cross-talk pathways involving mitochondrial damage, endoplasmic reticulum stress, and lysosomal injury is discussed.

## 1. Introduction

Patients with bacteremia may develop moderate or severe sepsis or life-threatening septic shock following multiple organ failure or multiple organ dysfunction syndrome (MOF/MODS) [1,2,3]. Patients with MOF/MODS have a 25–50% higher mortality rate than those without MOF/MODS [4,5]. Although a variety of strategies for managing sepsis have been developed [3,6], it is difficult to prevent because of its complex pathogenic mechanisms, which generally involve severe inflammation and cell death. Enterotoxins—chromosomally encoded exotoxins that are originally defined by their cytopathic effects—are generally produced from pathogenic bacterial organisms, such as *Escherichia coli* (*E. coli*) (alpha-hemolysin (HlyA), Shiga toxins (Stxs), cytotoxic necrotizing factors 1 (CNF1), heat-labile enterotoxins (LT))[7,8], *Staphylococcus aureus* (*S. aureus*)(staphylococcal enterotoxins B (SEB), staphylococcal alpha-toxins, Panton-Valentine leukocidin (PVL)[7,9], *Streptococcus pneumoniae* (*S. pneumoniae*), (streptolysin and pneumolysin)[7,10,11], *Clostridium perfringens* (*Clostridium perfringens* enterotoxins)[12], *Vibrio cholerae* (cholera toxins)[13], and *Shigella dysenteriae* (Stxs)[14]. In addition to inflammatory activation, these enterotoxins are cytopathic to host cells through lytic or non-lytic mechanisms by inducing necrosis or apoptosis, respectively. Apoptosis, also called programmed cell death, induced by bacterial infection is widely under investigation [15,16,17]. Extrinsic and intrinsic pathways of apoptosis involving intracellular organelle dysfunction and caspase cascade activation are regulated for bacterial enterotoxin-induced pro-apoptotic signaling. In addition to apoptosis, cytopathic studies have shown that several enterotoxins, including hemolysin, staphylococcal alpha-toxin, pneumolysin, and streptolysin-O, usually cause cell death by altering the apical membrane permeability of the targeting cells [7]. These cytopathic enterotoxins are pore-forming toxins (PFTs), also defined as cytolysins. After these cytolysins bind to a host cell membrane, the mechanism for pore formation involves the insertion of a number of water-soluble single-chain polypeptides into the membrane bi-layer and the formation of hydrophilic transmembrane pores [7,18]. The generation of hydrophilic transmembrane pores, which induces necrotic lysis or permeabilization of host cells or intracellular organelles during infection, is pathogenic for disease development via the disruption of infected tissues/cells and the induction of local and/or systemic immunosuppression. For microbial pathogenesis, somatic cell death and immune cell death are required for bacterial invasion and immune escape, respectively.

## 2. Apoptotic Cell Death

Apoptosis, also called programmed cell death, is generally involved in bacterial infection and pathogenesis [15,16,17]. During bacterial infection, virulent factors (mostly enterotoxins) are produced and secreted from pathogens and trigger apoptotic signals. In general, cells undergo apoptosis through two major pathways, the extrinsic pathway (the death receptor pathway) or the intrinsic pathway (the mitochondrial pathway)[19,20,21]. In addition to enterotoxins, the invasion and endocytosis of whole pathogens into the infected cells also cause apoptotic signaling through extrinsic and intrinsic pathways [15]. It is speculated that blockage of this apoptotic signaling may confer protection against bacterial infection-induced sepsis [20,21].

### 2.1. Extrinsic (Death Receptor-mediated) and Intrinsic (Mitochondria-Regulated) Pathways of Apoptosis

Extrinsic pathways are generally initiated by the activation of death receptors through the interaction between their natural ligands or by inducing death receptor clusterization. Death receptors are cell surface receptors that belong to the tumor necrosis factor (TNF) super family and interact with their ligands to form death receptor complexes, including Fas (CD95/Apo1)/Fas Ligand (CD95 ligand)[22], TNF receptor 1 (p55)/TNF and lymphotoxin [23], TRAMP (WSL-1/Apo3/DR3/LARD)/TWEAK (Apo3 ligand)[24], TRAIL-R1 (DR4)/TRAIL (Apo2 ligand)[25], and TRAIL-R2 (DR5/Apo2/KILLER)/TRAIL [26]. Upon extrinsic activation, the intracellular death domain (DD) of death receptors associates with an adaptor protein called Fas-associated death domain (FADD) directly or indirectly via the TNF receptor-associated death domain [26]. The death receptor associated intracellular FADD interacts with pro-caspase-8, a typical initial caspase, to form a death-inducing signaling complex required for caspase-8 activation [26]. During the process of apoptosis, there is, in general, a reduction of mitochondrial transmembrane potential followed by the release of cytochrome *c*, which binds to Apaf-1 and promotes caspase-9 and caspase-3 activation [27,28]. The central role of mitochondria in apoptosis is proposed to be via an intrinsic pathway. Pro-apoptotic Bax and Bid, the members of the Bcl-2 family with pro-apoptotic roles, translocate to the mitochondria and disrupt the membrane integrity, resulting in cytochrome *c* release from mitochondria to cytoplasm [29,30,31]. The induction of mitochondrial transmembrane permeabilization (MTP) resulted from Bax or truncated Bid (activated by caspase-8 from the extrinsic pathway) forms pores in the outer membrane directly or by interacting with the permeability of the transition pore complex [30,31]. In contrast, anti-apoptotic Bcl-2 and Bcl-xL protect these effects by maintaining the MTP through the inhibition of Bax or other pro-apoptotic factors [29]. The loss of balance of Bcl-2/Bax is believed to contribute to the progression of apoptosis.

### 2.2. Endoplasmic Reticulum Stress-Mediated Apoptosis

Stress on the endoplasmic reticulum (ER), which is the site of protein synthesis, modification, and folding, can be caused by multiple insults, such as the inhibition of glycosylation, the reduction of disulfide bonds, calcium depletion from the ER lumen, impairment of protein transport to the Golgi, and expression of mutated proteins in the ER, which can trigger an unfolded protein response (UPR) following ER stress [32,33,34]. These events enhance protein folding and degradation within the ER and down-regulate protein synthesis until cells have recovered from the ER stress. However, prolonged ER stress may eventually cause apoptosis, and calcium homeostasis and the UPR cannot be restored. Several apoptotic signaling pathways have been demonstrated in ER stress-induced apoptosis [32]. The ER stress-induced C/EBP homologous protein, a transcription factor that suppresses the expression of anti-apoptotic protein Bcl-2 and increases ROS production, is involved in apoptosis through the mitochondrial pathway [33]. ER stress-activated c-Jun N-terminal kinase is also involved [34]. Calpain, a calcium-dependent protease, generally causes the activation of human caspase-4, a specific ER stress-activated caspase with a 48% sequence homology to murine caspase-12 [35,36]. Notably, caspase-4 triggers apoptotic pathways, dependent or independent of caspase-9 and caspase-3 activation [32]. Furthermore, the activation of caspase-2, -3, -7, -8, and -9 have also been reported in ER stress-induced apoptosis [37,38,39]. However, the role of caspase cascade activation is still controversial, owing to differences in ER stressor stimulation and cell type dependence.

### 2.3. Lysosomal Pathway of Apoptotic Signaling

The lysosome, an acidic organelle, plays a pivotal role in apoptosis and necrosis caused by various stimuli, including oxidative stress, TNF-α, sphingosine, p53, and staurosporine [40,41]. However, the precise lysosomal pathway in ER stress-induced apoptosis remains unclear. Apoptotic stimuli cause lysosomal membrane permeabilization through a variety of regulatory factors, such as calcium, ROS, ceramide, sphingosine, phospholipase, Bax, Bim, Bid, and caspase [41,42]. The lysosomal proteolytic enzymes, mainly proteases of the cathepsin family, translocate to the cytosol. Once in the cytosol, cathepsin B and cathepsin D are the major mediators triggering apoptotic and necrotic pathways, which involve Bid truncation, caspase activation, and mitochondrial damage [43]. Under apoptotic stress, both ER stress and lysosomal and mitochondrial destabilization may contribute to the initiation stage of apoptosis.

## 3. Necrotic Cell Death

Necrotic cell death is also caused by a variety of extracellular and intracellular factors, such as bacterial and viral infections, toxins, or trauma [44]. Numerous intracellular molecules are involved in necrotic cell death, including ROS, Ca^2+^, calpains, cathepsins, phospholipases, and ceramide [45]. In contrast with apoptosis, necrosis is characterized by severe pathophysiological changes, including mitochondrial swelling, rapid loss of plasma membrane integrity, ultimate leakage of cellular contents, and a lack of typical apoptotic features, such as internucleosomal DNA cleavage and nuclear condensation. Necrotic cell death is identified as caspase-independent cell death [46]. Exposing the release content proteins triggers acute inflammation and causes pathogenic to the host.

Necrotic cell death is also induced through death receptor-mediated signaling. Activation of the death receptors, including Fas and TNF-α receptors, induces a “prototypic” apoptotic pathway through FADD. Under apoptosis-deficient conditions, stimulation with FasL or TNF-α still induces cell death with the morphological features of necrosis [47]. Notably, necrostatin-1, an inhibitor of RIP1, blocks Fas- and TNF-α receptor-mediated necrotic cell death, namely necroptosis [48]. In addition to death receptor pathways, the signaling of pathogen recognition receptors such as the Toll-like receptor (TLR) 3 [49] and TLR4 [50] and extensive DNA damage [51] can also lead to necrotic cell death.

## 4. Cytotoxic Enterotoxins

### 4.1. Cytotoxicity of *S. aureus*

*S. aureus*, a Gram-positive bacterium, causes a variety of diseases ranging from minor skin infections to life-threatening conditions such as staphylococcal septic shock [9]. Cell wall components and secreted enterotoxins have been shown to be inflammatory, cytotoxic, and septic mediators. These factors may be recognized by the innate immune system via multiple manners [52]. To date, it is believed that mechanisms that lead to staphylococcal septic shock are probably multifactorial but include immunogenic and cytotoxic injuries.

Staphylococcal superantigens, a large family of exotoxins, are powerful microbial toxins that activate the immune system by binding to class II major histocompatibility complex and T-cell receptor molecules without antigen processing. There are 17 well-characterized, serologically distinct staphylococcal superantigens made by *S. aureus*: TSS toxin-1; staphylococcal enterotoxins (SEs) A, B, C (multiple minor variant forms exist), D, E, and I; and SE-like G, H, J, K, L, M, N, O, P, and Q [53]. Superantigen SEB, a staphylococcal pyrogenic exotoxin, causes cellular cytotoxicity by inducing cell activation followed by the induction of inflammatory cytokines including TNF-α, interleukin (IL)-2, IL-6, IL-10, and interferon-γ, and chemokines including monocyte chemoattractant protein 1 regulated on activation, and normal T-cell expressed and secreted proteins [54,55,56,57,58]. Further study showed that TNF-α plays an initial role in inflammation on T cell-mediated toxicity in SEB-induced septic shock, whereas anti-TNF-α-neutralizing monoclonal antibody conferred protection against SEB [59]. Interestingly, the major producer cells of TNF-α are CD3 positive splenic T cells and peritoneal cells. In addition to the pathogenic role of SEB *in vivo*, *in vitro* studies showed that superantigens are enabled to induce transcriptional activation of the TNF in T cells and monocytes [60,61]. In SEB-induced septic pathogenesis, T cells are the major cause of shock syndrome [54,59]. Furthermore, SEB may cause CD4 positive cells undergoing activation-induced cell death both *in vitro* and *in vivo* [62,63,64,65]. Izquierdo and colleagues [66] found that the blockade of caspase activation inhibits SEB-induced thymocyte apoptosis. Current studies show that the induction of caspase-3-mediated apoptosis in SEB-exposed renal proximal tubule epithelial cells involves the signaling of Rho family proteins [67]. The molecular mechanism for SEB-induced cell apoptosis needs further investigation.

The staphylococcal alpha-toxin, a water-soluble single-chain cytopathic toxin produced by *S. aureus*, was the first staphylococcal exotoxin to be identified as a PFT [68]. It contains high-affinity structures that interact with a variety of cells, including rabbit erythrocytes, human platelets, monocytes and endothelial cells. After it binds onto a membrane, the generation of ring-structured hexamers is present as a pore. These pores trigger cellular responses via a Ca^2+^ influx to lyse and permeabilize cell membranes. Staphylococcal alpha-toxin-exposed keratinocytes and T cells show the depletion of cellular ATP followed by the degradation of internucleosomal DNA [69,70]. To characterize the type of cell death, Bantel and colleagues [71] demonstrated that a staphylococcal alpha-toxin induces cytochrome *c* release in a Bcl-2-controlled manner. They further demonstrate that staphylococcal alpha-toxin causes an intrinsic mitochondrial pathway, which involves caspase-9 and caspase-3 cascade activation independently of the death receptor pathway. However, Essmann and colleagues [72] provided data suggesting that although pan-caspase inhibitor zVAD-fmk or overexpression of Bcl-2 completely decreased staphylococcal alpha-toxin-induced caspase activation and internucleosomal DNA fragmentation, they did not significantly inhibit cell death of T or breast carcinoma cells. Notably, electron microscopy demonstrated that a staphylococcal alpha-toxin induces necrotic cell death, which is characterized by cell swelling and cytoplasmic vacuolation. In addition to apoptosis and necrosis, the staphylococcal alpha-toxin may facilitate death receptor-mediated caspase-8 activation and apoptosis through TNF-α upregulation [73]. Taken together, these data suggest that a staphylococcal alpha-toxin induces cell death in a cell type- and dose-dependent manner, including apoptosis and necrosis. Mitochondrial dysfunction caused by a staphylococcal alpha-toxin is usually involved either in apoptosis or necrotic cell death. In addition to the involvement of mitochondrial injury, Bernheimer and Schwartz [74] identified that bacterial toxins, including the staphylococcal alpha-toxin, the *Clostridium perfringens* alpha-toxin, and streptolysins O and S, affected lysosomes. It is notable that lysosomotropic agents NH_4_Cl, chloroquine, and monensin effectively abrogated staphylococcal alpha-toxin-induced internucleosomal DNA fragmentation [75]. The involvement of lysosomal pathway in staphylococcal alpha-toxin-induced apoptosis and necrotic cell death therefore needs further investigation.

Current reports demonstrate the emerging role of PVL, a key PFT, for *S. aureus*-induced rapidly progressive, hemorrhagic, necrotizing pneumonia [76,77]. These pneumonias occurred in younger patients, and the presence of leucopenia and the mortality rate represented 79% and 75% of cases, respectively [77]. PVL-producing *S. aureus* strains cause polymorphonuclear cell death by necrosis or by apoptosis [78,79,80]. The PVL concentration determines the type of cell death. For cell apoptosis, PVL causes mitochondrial damage followed by the activation of caspase-9 and caspase-3 independently of pro-apoptotic Bax activation. Notably, PVL directly inserts into the outer membrane of the mitochondria and induces the release of cytochrome *c* and Smac/DIABLO through pore formation [78].

### 4.2. Cytotoxicity of *E. coli*

**Figure 1:**
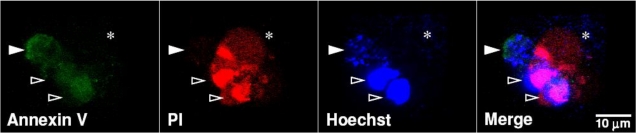
*In vitro* infection of Uropathogenic *E. coli* (UPEC) causes apoptotic and necrotic cell death in human renal proximal tubular epithelial cells. Human renal proximal tubular epithelial cells (HK-2) were infected with or without clinical isolates of UPEC (MOI = 1) for 12 h. Annexin V (*green*), propidium iodide (PI, *red*), and Hoechst 33258 (*blue*) were used for apoptotic (solid arrowheads), necrotic (empty arrowheads), and nucleic acid staining, respectively, and analyzed by confocal microscopy. Stars indicate *E. coli*.

Uropathogenic *E. coli* (UPEC) frequently accounts for the community-acquired urinary tract infection (UTI), in both cystitis and pyelonephritis. Severe UTI of type 1-piliated UPEC is the common cause of bacteremia, leading to a health-threatening concern [81]. UPEC can invade epithelial cells of the urinary tract, and these bacterial inclusions can form intracellular bacterial communities to invade the host cell [82]. After the infection of type 1-piliated UPEC, exfoliation of the host bladder epithelial cells shows a rapid apoptotic mechanism involving caspase activation and internucleosomal DNA fragmentation [83]. Diffusely adhering *E. coli* strains expressing adhesins of the Afa/Dr family bind to epithelial cells and express a functional hemolysin that promotes cell apoptosis or necrosis [84]. In UPEC-infected human renal proximal tubular epithelial cells, either apoptosis or necrosis is observed (Figure 1). This indicates that *E. coli* has multiple cytotoxic effects depending on the cell types of infection and the various virulent actions.

Among the numerous toxins produced by *E. coli*, HlyA, which belongs to a member of the repeats-in-toxin family of PFTs, causes cell apoptosis or necrosis [85]. HlyA possess cytotoxicity in different cells such as erythrocytes [86], granulocytes [87,88,89,90], monocytes [91], endothelial cells [92], renal tubular epithelial cells [93], and T cells [94]. The toxic effect of HlyA is due to the generation of the transmembrane pore after its insertion into the plasma membrane [86]. Increased permeability of cations and water leads to cell death. In human monocyte-derived macrophages and J774 cells (the murine macrophage cell line), HlyA is required to induce necrosis and apoptosis [95]. Moreover, HlyA induces neutrophil apoptosis (at lower *E. coli* titers) and necrosis/lysis (at lower *E. coli* titers) *in vitro*. In a rat pneumonia model, HlyA induces neutrophil necrosis/lysis rather than apoptosis [96].

Besides HlyA, several serotypes of *E. coli* express Stxs to induce apoptosis in several cell types including epithelial [97,98,99,100], endothelial [101,102,103,104], monocytic [105,106,107], lymphoid [108], and neuronal [109]. Stxs belong to the Shiga toxin family, which is made up of a group of structurally and functionally related exotoxins produced by the *Shigella dysenteriae* serotype 1 and enterohemorrhagic *E. coli* (EHEC). EHEC infection causes diarrhea, hemorrhagic colitis, and hemolytic uremic syndrome (HUS)[110]. HUS is the most common cause of acute renal failure in children [111]. Stxs induce apoptosis and contribute to the HUS-associated injury of renal endothelium [112]. Stxs are AB chain toxins in which the B chain binds to the cell surface receptor and mediates the subsequent endocytosis of the toxin, and the A chain acts as a protein synthesis inhibitor through its RNA *N*-glycosidase activity to inactivate ribosomes enzymatically [113]. The apoptotic pathways activated by Stxs are involved in the initial induction of ER stress and the subsequent calcium release to activate calcium-dependent cysteine protease calpain to further activate downstream caspase-8 and caspase-3. The increased expression of the death receptor 5 and TRAIL were also addressed [107]. Of note, the caspases activated by Stxs, including caspase-2, -3, -6, -8, and -9, differ depending on the different cell types [102,105,107,114,115]. The participation of Bcl-2 family proteins is also reported in Stxs-induced apoptosis, including truncated Bid and Bax to initiate a mitochondrial pathway [97,107]. The expression of Mcl-1 is down-regulated upon stimulation of Stxs [101], and Bcl-2 confers protection against Stxs [97,106,116]. In addition, the intracellular trafficking of Stxs has been extensively studied because the A chain of Stxs has to enter the cytoplasm to execute its cytotoxicity by inhibiting protein synthesis. After internalization, Stxs escape from the endosome and undergo retrograde delivery to the Golgi apparatus, then to the ER membranes, and finally to the cytosol, by using ER-associated degradation machinery [117,118]. Inhibiting the transportation of Stxs from the endosome to the Golgi apparatus has been implicated to prevent Stxs-induced apoptosis [115,119]. An old theory of using protein toxins to induce apoptosis in cancer cells is still applied in some pioneering studies that have been recently reviewed [113].

*E. coli* CNF1 activates proteins belonging to the Rho family of small GTP-binding proteins, which induce the rearrangement of the actin cytoskeleton to cause a phagocytic-like behavior called macropinocytosis [120,121,122]. Previous studies have demonstrated that these virulence factors can directly induce apoptosis or necrosis, depending on concentration, in a variety of cell types [123,124]. UPEC CNF1 activates the Cdc42 and Rac proteins to induce apoptosis in CNF1-intoxicated 5637 bladder epithelial cells [124]. In addition to cell death, the role of CNF in regulating cell fate is controversial. Previous reports [125,126] showed that the Rac-activating toxin CNF1 causes aneuploidy and multinucleation and prevents the ultraviolet-B-induced apoptosis in epithelial cells through the Akt/IkappaB kinase-regulated Bcl-2 pathway. The contribution of cytotoxic CNF1 in the pathogenesis of *E. coli* infection needs further investigation.

Heat-labile enterotoxin (LT) is an important diarrheal agent produced by *E. coli* [127,128]. Similar to Stxs, LT is an AB toxin, which consists of a B pentamer and a monomeric A subunit. Nontoxic B pentamer adheres to the oligosaccharide portion of ganglioside GM1 on the surface of eukaryotic cells and mediates endocytosis of the toxin. The monomeric A subunit possesses ADP-ribosyltransferase activity and activates adenylate cyclase to elevate cyclic AMP levels. A constitutive increase of intracellular cylic AMP leads to an imbalance of electrolytes and results in diarrhea [127,128,129]. Previous reports have shown that LT has a variety of adjuvant activities, such as increasing the production of antibodies, activating B cells and CD4^+^ cells, and suppressing the number of CD8^+^ cells [130,131,132,133,134,135]. LT is further divided into type I (LT-1) and type II (LT-IIa and LT-IIb), depending on its distinct receptor binding specificity and immunomodulatory abilities [128]. LT-IIa, but not LT-IIb, induces apoptosis in CD8^+^ T cells *in vitro* [136]. *In vivo* treatment with LT-1 increases the serum levels of corticosterone to induce apoptosis of immature T and B cell populations by a Fas- and TNFR1-independent, caspase-dependent, and apoptosis-inducing factor-independent mechanism [137,138]. LT-1-induced *in vivo* apoptosis requires intact ADP-ribosyltransferase activity and partially protects by overexpression of Bcl-2 [137]. *In vitro* studies have shown that the B subunit of LT induced selective loss of CD8^+^ T cells by triggering apoptosis through c-Myc, NF-κB, and caspase-dependent and -independent pathways [139,140,141,142]. The immunomodulatory abilities of the LT B subunit relied on its binding to GM1 because non-binding mutants did not have pro-apoptotic properties [130,131,132,139]. The mechanisms by which LT induces apoptosis *in vivo* and *in vitro* and in different subsets of lymphoid cells might be quiet different [137,138,143].

### 4.3. Cytotoxicity of *V. cholerae*

*Vibrio cholera,* a Gram-negative bacterium, is the causative agent of severe diarrhea cholera, which leads to dehydration and metabolic acidosis [144,145]. Regardless of the fact that there are at least 200 serogroups of *V. cholera*, only the O1 and O139 serogroups have been associated with cholera [146]. *V. cholera* secretes a variety of toxins, including cholera enterotoxin, zonula occludens toxin, accessory cholera enterotoxin, hemolysin/cytolysin, Shiga-like toxin, heat-stable enterotoxin, new cholera toxin, sodium channel inhibitor, and thermostable direct hemolysin, all of which have deleterious effects on eukaryotic cells [147]. Because of the massive, dehydrating diarrhea caused by cholera enterotoxin, also called cholera toxin (CT), it plays a pivotal role in cholera diseases [148]. CT is an oligomeric complex composed of one A subunit (CTA) and five B subunits (CTB)[149]. The A subunit is responsible for the direct toxic activity that is due to its elevation of cAMP in cells by ADP-ribosylation of Gsα protein, a regulator of adenylate cyclase, and the B subunit that serves to bind the holotoxin to the receptor, the ganglioside GM1 [150,151,152,153]. CT and its purified CTB process the immunosuppressive effect, which owes not only to proliferation inhibition but also to apoptosis induction in lymphoid cells. Previous studies have shown that no matter whether CT or CTB preferentially induced apoptosis in CD8+ T cell population, the mechanism is still unclear [136,154,155,156]. Besides, CT also stimulates apoptosis in human lung cancer cell lines and the murine myelomonocytic leukemia cell line, WEHI-3B, but down-regulation of Bcl-2 and inhibition of apoptosis developed in a CT resistant clone [157,158]. However, the mechanism of CT-induced apoptosis remains largely unknown and requires further investigation.

## 5. Concluding Remarks

The effects of cytopathic enterotoxins are pro-apoptotic as well as necrotic-like, depending on the types of cells and the dosages of stimulation. In general, there are two or more phenomena under cytolysin stimulation. Apoptotic signaling caused by enterotoxins is diverse because several intracellular organelles are generally involved. Upon the formation of pores following PFT stimulation, supporting the membrane integrity is important for cell survival. Statins, the cholesterol-lowering agents, have recently been used to inhibit pneumococcal cholesterol-dependent cytolysin pneumolysin-induced cytotoxicity on endothelia and epithelia [159]. Inhibiting cell death via the stimulation of virulent enterotoxins is a key strategy for treating patients with infectious diseases [17,85].
